# Efficacy and safety of once-weekly insulin versus once-daily insulin in patients with type 1 and type 2 diabetes mellitus: an updated meta-analysis of randomized controlled trials

**DOI:** 10.3389/fendo.2024.1459127

**Published:** 2024-11-19

**Authors:** Mei Xue, Pan Shen, Jun Tang, Xuan Deng, Zhe Dai

**Affiliations:** ^1^ Department of Endocrinology, Zhongnan Hospital of Wuhan University, Wuhan, China; ^2^ Department of Dermatology, Tongji Hospital, Tongji Medical College of Huazhong University of Science and Technology, Wuhan, China; ^3^ Department of Nephrology, Zhongnan Hospital of Wuhan University, Wuhan, China

**Keywords:** once-weekly insulin, once-daily insulin, diabetes mellitus, glycosylated hemoglobin (HbA1c), hypoglycemia

## Abstract

**Background:**

This meta-analysis was performed to obtain a comprehensive overview of the differences between once-weekly basal insulin (including icodec and basal insulin Fc) and once-daily basal insulin (including glargine and degludec) in patients with type 1 and type 2 diabetes mellitus.

**Methods:**

PubMed, EMBASE, and Cochrane Library were systematically searched for eligible studies up to 2 January 2024.

**Results:**

A total of 12 studies were included, comprising 5,895 patients, with 3,104 (52.7%) using once-weekly insulin and 2,791 (47.3%) using once-daily insulin. In the pooled data, glycated hemoglobin (HbA1c) change from baseline [mean difference (MD) -0.11%; 95% confidence interval (CI) -0.20 to -0.01%] and the odds of achieving an end-of-trial HbA1c <7% (OR 1.41, 95% CI 1.13, 1.77) demonstrated a significantly good glycemic control in the once-weekly insulin group, especially in insulin-naïve type 2 diabetics or patients using icodec. Body weight increase for once-weekly insulin was 0.43 kg compared to controls (95% CI 0.09 to 0.76 kg). In addition, once-weekly insulin was correlated with a higher risk of level 1 hypoglycemia (OR 1.42, 95% CI 1.26 to 1.6). There was no significant difference in fasting plasma glucose (MD 2.46 mg/dL; 95% CI -2.60 to 7.52 mg/dL), time in range (MD 2.03%; 95% CI -0.50 to 4.56%), and level 2 or 3 hypoglycemic events (OR 1.19; 95% CI 0.93 to 1.53).

**Conclusions:**

Once-weekly basal insulin is safe and effective in modestly reducing HbA1c with similar level 2 or 3 hypoglycemic events compared to once-daily insulin, although the risk of level 1 hypoglycemia and weight gain was slightly increased.

**Systematic review registration:**

https://www.crd.york.ac.uk/PROSPERO, Identifier CRD42024496812.

## 1 Introduction

Diabetes has become a global public health concern, and it is well known that both type 1 diabetes and type 2 diabetes require effective blood glucose control to prevent the development of micro- and macrovascular complications. Insulin was invented over a hundred years ago, and insulin therapy plays an important role in the treatment of subjects with diabetes who have absolute or relative insulin deficiency (1, 2). Long-acting basal insulin provides basal support for patients with type 1 diabetes, which is an indispensable component of basal-bolus therapy. Despite the wide variety of hypoglycemic medications available, subjects with type 2 diabetes will eventually require insulin therapy with basal insulin being usually used as the initiation of insulin treatment (3). However, the risk of hypoglycemia, other side effects such as excessive weight gain, and the fear of daily injections can limit insulin use in patients, and these shortcomings have prompted researchers to refine insulin formulations (4).

Currently, novel basal insulin analogs have been designed for once-weekly subcutaneous administration that may improve treatment acceptance and adherence. Icodec and basal insulin Fc (BIF, also known as insulin efsitora alfa or LY3209590) are the two most advanced once-weekly basal insulins for the treatment of patients with type 1 or type 2 diabetes (5). Icodec increases reversible binding to albumin and reduces insulin receptor affinity by acylation with a C20 fatty diacid side chain and specific amino acid substitutions, which has a plasma half-life of 196 h in humans and achieves a steady state after 3–4 weekly injections (5, 6). BIF is a fusion protein combining a single-chain insulin variant with a human IgG2 Fc domain. BIF has a half-life of approximately 17 days, and the seven-point glucose profiles remain constant, similar to once-daily insulin (5, 7).

Compared to regular once-daily insulin, these new ultra-long-acting insulin analogs can reduce the injection burden by 85%, thereby improving patient treatment compliance (8). Several phase 2 and 3 randomized controlled trials (RCTs) have recently been published, which investigated the efficacy and safety of novel once-weekly basal insulin analogs in blood glucose control compared to once-daily basal insulin (degludec, glargine U100, or glargine U300) (9–12). Particularly, the phase 3a ONWARDS 1–6 trials in adults with type 1 and type 2 diabetes have now been completed and reported, making icodec a brighter prospect for glycemic control (13, 14). Previous meta-analyses focused on their use only in patients with type 2 diabetes and demonstrated that icodec and BIF provided effective and safe blood glucose control comparable to once-daily insulin (15–19). Recently, data from clinical RCTs in patients with type 1 diabetes have become available, and it is therefore necessary to summarize the evidence from these published trials for the treatment of both type 1 and type 2 diabetes mellitus.

This paper reports the results of a comprehensive set of patient-level meta-analyses that were performed to compare once-weekly basal insulin and once-daily basal insulin for the primary endpoint (HbA1c) and secondary endpoints [FPG, TIR, number of patients achieving HbA1c of <7%, body weight, hypoglycemia (level 1), any adverse event, serious adverse events, severe adverse events, any adverse event probably or possibly related to basal insulin, injection-site reactions, and hypersensitivity events] in diverse populations across the spectrum of diabetes.

## 2 Methods

This meta-analysis followed the Preferred Reporting Items for Systematic Reviews and Meta-Analysis (PRISMA) guideline. Our protocol was registered on PROSPERO [CRD42024496812] with the title “Efficacy and safety of once-weekly insulin versus once-daily insulin in patients with type 1 and type 2 diabetes mellitus: A meta-analysis of randomized controlled trials”.

### 2.1 Search strategy

PubMed, EMBASE, and Cochrane Library were comprehensively searched from inception to 2 January 2024. The detailed search strategy is presented in **Supplementary Tables 1**–**3**. Two distinct and independent investigators screened and reviewed each abstract and/or full-text manuscript, and discrepancies were resolved with a third author. Only studies published in English were included.

### 2.2 Eligibility criteria

Studies were included if they fulfilled the following criteria: (1) Participants: patients who were diagnosed with type 1 or type 2 diabetes; (2) Intervention: once-weekly basal insulin, including icodec and BIF; (3) Comparison: once-daily basal insulin, including glargine and degludec; (4) Outcome: efficacy outcomes (HbA1c, FPG, TIR, body weight, and achieving HbA1c<7.0% at the end of trial) and safety outcomes (hypoglycemia and other related adverse events); (5) Study: RCTs. We excluded meta-analyses and systematic reviews, comments, editorials, pharmacokinetic/pharmacodynamic studies, and studies not reporting the outcome of interest.

### 2.3 Data extraction

Relevant data from eligible trials were independently extracted by two investigators. A third author was consulted to resolve discrepancies. Briefly, we recorded the baseline characteristics of the RCTs including the name of the first author, publication year, phase of the RCT, type of diabetes, trial duration, follow-up duration, background insulin therapy, number of participants, intervention measures, age, sex ratio, diabetes duration, HbA1c, FPG, body weight, and body mass index (BMI). The primary outcome of the quantitative meta-analysis was HbA1c. Secondary outcomes were FPG, TIR, body weight, number of patients achieving HbA1c of <7%, hypoglycemia (level 1), clinically significant (level 2) or severe (level 3) hypoglycemia, any adverse event, serious adverse events, severe adverse events, any adverse event probably or possibly related to basal insulin, injection site reaction, and hypersensitivity events.

### 2.4 Quality assessment

Two investigators evaluated the quality of each trial using the Cochrane Risk of Bias Tool in seven domains: random sequence generation, allocation concealment, blinding of participants and personnel, blinding of outcome assessment, incomplete outcome data, selective reporting, and other biases. Items were scored as low, high, or unclear risk of bias. A third author was consulted to resolve discrepancies. Finally, publication bias was visually assessed using a funnel plot.

### 2.5 Statistical analysis

All statistical analyses were performed by RevMan5.4 software (The Nordic Cochrane Centre, The Cochrane Collaboration). The odds ratio (OR) was used to evaluate dichotomous variables, and the mean difference (MD) was used to evaluate continuous variables, with a 95% confidence interval (CI). The heterogeneity was analyzed with the *I* ^2^ and *Q* tests. When the *p*-value ≥ 0.1 for the *Q* test or *I* ^2^ ≤ 50%, a fixed‐effect model was used. The *p*-value < 0.1 for the *Q* test or *I* ^2^ > 50% was considered significant heterogeneity between studies, and then a random‐effect model was applied. Subgroup analyses were conducted according to the different types of participants (insulin-treated type 1 diabetics vs. insulin-naïve type 2 diabetics vs. previously insulin-treated type 2 diabetics), types of once-weekly insulin (icodec vs. BIF), trial duration (≤26 weeks vs. >26 weeks), diabetes duration (below median duration vs. above median duration), and types of once-daily insulin (degludec vs. glargine).

## 3 Results

### 3.1 Search results

**Figure 1:**
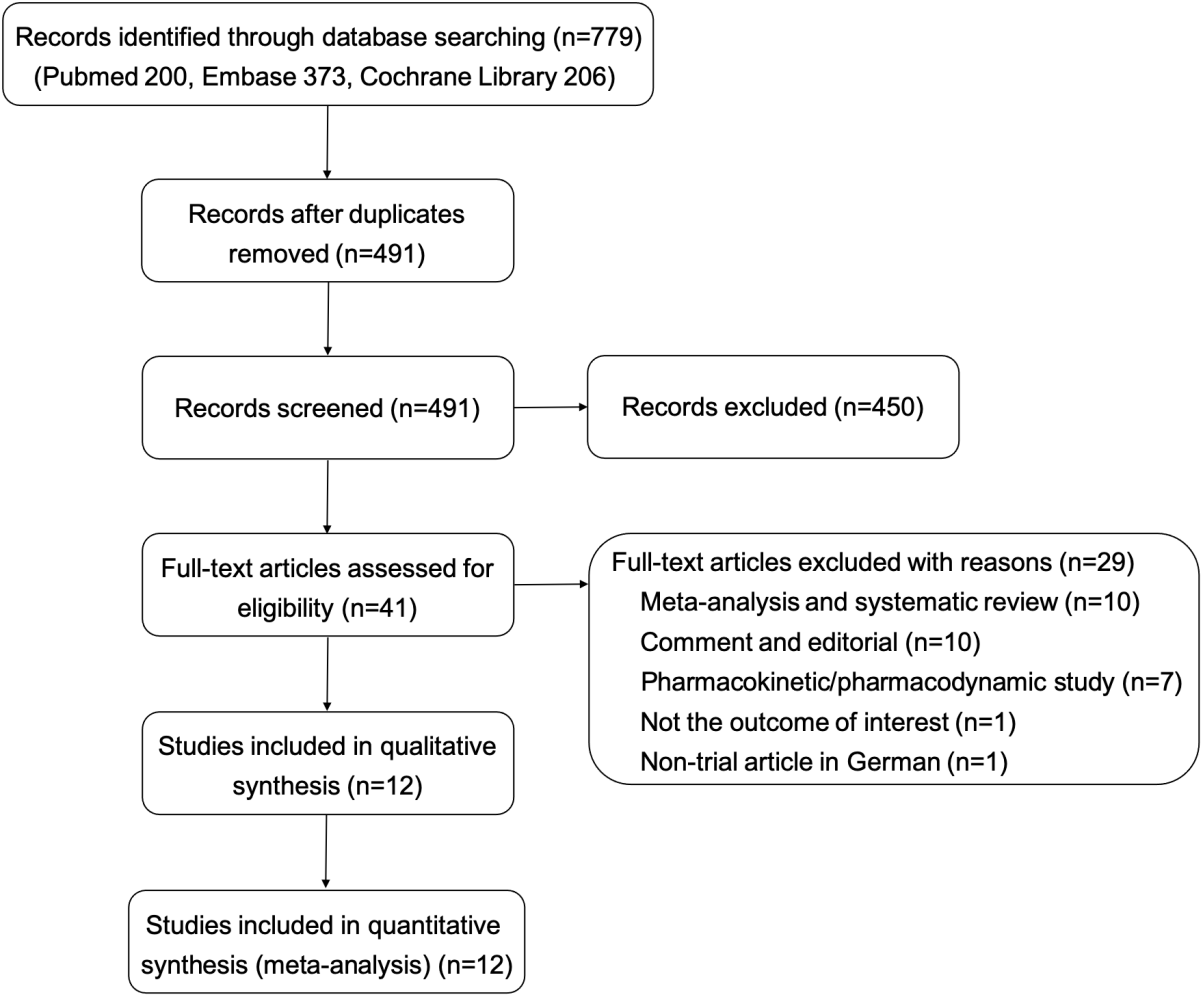
Flow diagram of study search, screening, and selection.

In total, 779 references were identified through the search strategy outlined, of which 288 were removed as duplicates. Subsequently, 491 records were screened by title and abstract, of which 450 were excluded. After reading the full text of 41 studies, 12 eligible studies (9–12, 20–27) were included in the analysis. Details of the search and selection process are presented in **Figure 1**.

### 3.2 Study characteristics

A total of 5,895 patients from 12 studies reporting outcomes of interest met the eligibility criteria (**Table 1**). Of these, 3,104 (52.7%) received once-weekly insulin and 2,791 (47.3%) received once-daily insulin. The study population was divided into three categories: insulin-treated type 1 diabetes mellitus (T1DM) included two studies (16.7%), insulin-naïve type 2 diabetes mellitus (insulin-naïve T2DM) included six studies (50%), and previously insulin-treated T2DM (non-insulin-naïve T2DM) included four studies (33.3%). The types of once-weekly insulin used in these trials were icodec (nine studies, 75%) and BIF (three studies, 25%). The main characteristics of the included trials are presented in **Table 1**. A total of seven (75%) trials had a duration of less than or equal to 26 weeks, and three (25%) trials had a duration of more than 26 weeks. The mean age across all included participants ranged from 44.1 to 62.6 years, with a slight male (49%–75%) preponderance. The mean diabetes duration varied from 8.8 to 22.3 years. The mean baseline HbA1c of the participants reached 7.5% or higher (7.5%-8.96%) and the mean baseline FPG was greater than 140 mg/dL (141.2–185.7 mg/dL). The mean body weight of the diabetic patients at baseline ranged from 77.1 to 94.3 kg, with a BMI ranging from 26.2 to 33 kg/m^2^ (**Table 1**).

**Table 1:** Characteristics of the trials included in the meta-analysis.

Author Year	Type of diabetes	Trial duration (weeks)	Background insulin therapy	Number of participants		Intervention measures		Age (years)		Male patients (%)		Diabetes duration (years)		HbA1c (%)		FPG (mg/dL)		Body weight (kg)		BMI (kg/m^2^)	
				Once-weekly	Once-daily	Once-weekly	Once-daily	Once-weekly	Once-daily	Once-weekly	Once-daily	Once-weekly	Once-daily	Once-weekly	Once-daily	Once-weekly	Once-daily	Once-weekly	Once-daily	Once-weekly	Once-daily
Rosenstock 2020	Type 2	26	Insulin naïve	125	122	Icodec	Glargine U100	59.7 (8.2)	59.4 (9.5)	56	56.6	10.5 (8.4)	8.8 (6.1)	8.09 (0.7)	7.96 (0.65)	182 (42)	180 (42)	89.7 (16.5)	91.3 (15.7)	31.1 (4.9)	31.4 (4.4)
Lingvay 2021	Type 2	16	Insulin naïve	154	51	Icodec	Glargine U100	61.8 (8.4)	60.2 (8.1)	53.9	52.9	9.5 (5.6)	11.8 (6.8)	8.1 (0.8)	8.2 (0.8)	177 (33.3)	168 (42)	89.7 (16.6)	86.4 (17.1)	31.5 (4.5)	30.6 (4.7)
Bajaj 2021	Type 2	16	Basal insulin	104	50	Icodec	Glargine U100	62.3 (7.7)	60.5 (7.9)	75	66	15.2 (8)	14.8 (8.1)	7.8 (0.7)	7.9 (0.7)	143 (37.4)	148 (36)	NR	NR	29.6 (4.2)	30.3 (5)
Frias 2023	Type 2	32	Basal insulin	267	132	BIF	Degludec	59.9 (10.6)	60.8 (10)	49	51	15.1 (8)	14.6 (8.8)	8.1 (0.9)	8.1 (0.9)	141.2 (50.2)	144.5 (51)	89.4 (19.2)	87.1 (20.7)	32.5 (5.9)	31.8 (5.7)
Kazda 2023	Type 1	26	Basal-Bolus insulin	139	126	BIF	Degludec	45.5 (15.3)	47.4 (13.7)	61.9	61.9	22 (13.1)	22.3 (13.9)	7.5 (0.8)	7.5 (0.9)	165.4 (67.9)	159.3 (67.1)	81.3 (16)	82 (15.1)	27.5 (4)	27.2 (4.1)
Bue-Valleskey 2023	Type 2	26	Insulin naïve	143	135	BIF	Degludec	57.3 (9.7)	59.4 (9.1)	53.1	56.3	10.4 (6.8)	9.7 (6)	8.1 (0.8)	8 (0.8)	170.2 (42)	160.7 (36.7)	91 (20.8)	90.6 (19.6)	32.3 (5.4)	31.6 (5.5)
Philis- Tsimikas 2023	Type 2	26	Basal insulin	263	263	Icodec	Degludec	62.3 (9.8)	62.6 (8.4)	62	53	16.5 (8.4)	16.9 (7.9)	8.17 (0.77)	8.1 (0.77)	152.2 (47.5)	150.7 (40.9)	83.7 (18.4)	81.5 (17.1)	29.5 (5.2)	29.2 (4.9)
Mathieu 2023	Type 2	26	Basal-bolus insulin	291	291	Icodec	Glargine U100	59.7 (10.1)	59.9 (9.9)	53	52	18 (9.1)	16.3 (7.7)	8.29 (0.86)	8.31 (0.9)	167 (54)	173 (63)	85.5 (17.6)	83.1 (17.3)	30.5 (5)	30 (5)
Lingvay 2023	Type 2	26	Insulin naïve	294	294	Icodec	Degludec	58 (10)	59 (10)	62.9	62.6	10.3 (6.6)	11.1 (7.3)	8.55 (1.11)	8.48 (1.01)	187 (54)	176 (46)	85.8 (20.1)	83.2 (18.2)	29.9 (5.2)	29.2 (5.1)
Rosenstock 2023	Type 2	52	Insulin naïve	492	492	Icodec	Glargine U100	59.1 (10.1)	58.9 (9.9)	60	53.5	11.6 (6.7)	11.5 (6.8)	8.5 (1)	8.4 (1)	185.3 (49)	185.7 (51.7)	85.2 (17.7)	84.3 (17.6)	30 (4.8)	30.1 (5.1)
Bajaj 2023	Type 2	52	Insulin naïve	542	543	Icodec	Degludec/Glargine U100/Glargine U300	59.1 (10.8)	59.4 (10.2)	57	57.6	11.9 (6.9)	12 (7.6)	8.96 (1.6)	8.88 (1.5)	NR	NR	93.2 (22.5)	94.3 (21.5)	32.6 (7)	33 (6.9)
Russell-Jones 2023	Type 1	26	Basal-bolus insulin	290	292	Icodec	Degludec	44.1 (14.1)	44.3 (14.1)	57	59	20 (13.2)	19 (12.9)	7.59 (0.96)	7.63 (0.93)	179 (74)	172 (72)	78.6 (17.6)	77.1 (16.8)	26.8 (5)	26.2 (4.5)

Data are presented as mean (SD).

HbA1c, glycated hemoglobin A1c; BMI, body mass index; FPG, fasting plasma glucose; NR, not reported.

### 3.3 Quality assessment

**Figure 2:**
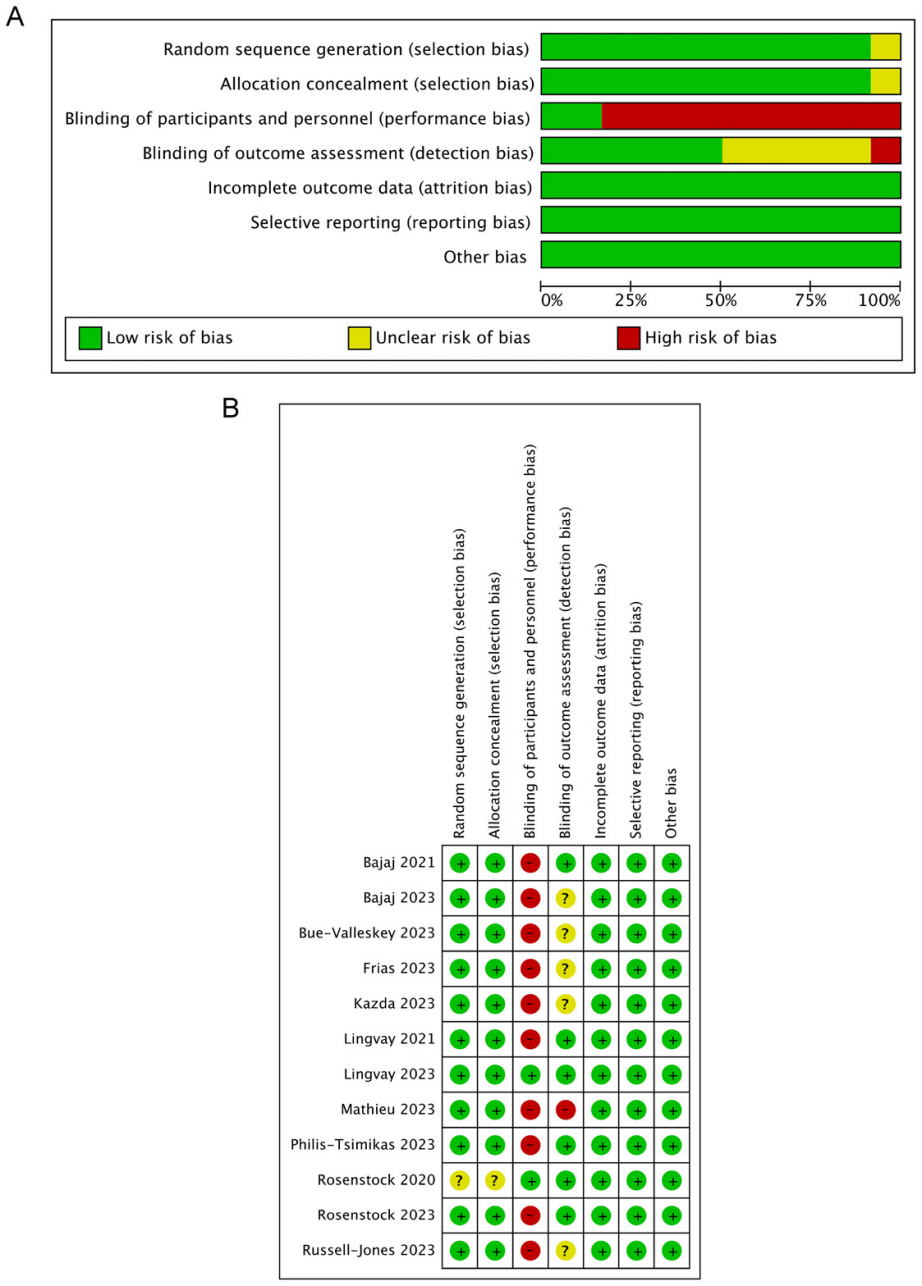
Risk of bias assessments of the included trials. **(A)** Summary of risk of bias across categories, presented as percentages. **(B)** Risk of bias graph for each study.

The risk of bias in the included trials is presented in **Figure 2**. In general, the included studies showed a low risk of selection, attrition, reporting, and other biases. Only 2 of the 12 studies were blinded to the participants and personnel, and the outcome assessment of six trials was blinded, showing a relatively high risk of performance and detection bias. In addition, funnel plots of the primary outcome showed visual symmetry of the scatter on both sides, indicating no prominent publication bias (**Supplementary Figure 1**).

### 3.4 Efficacy outcomes

**Figure 3:**
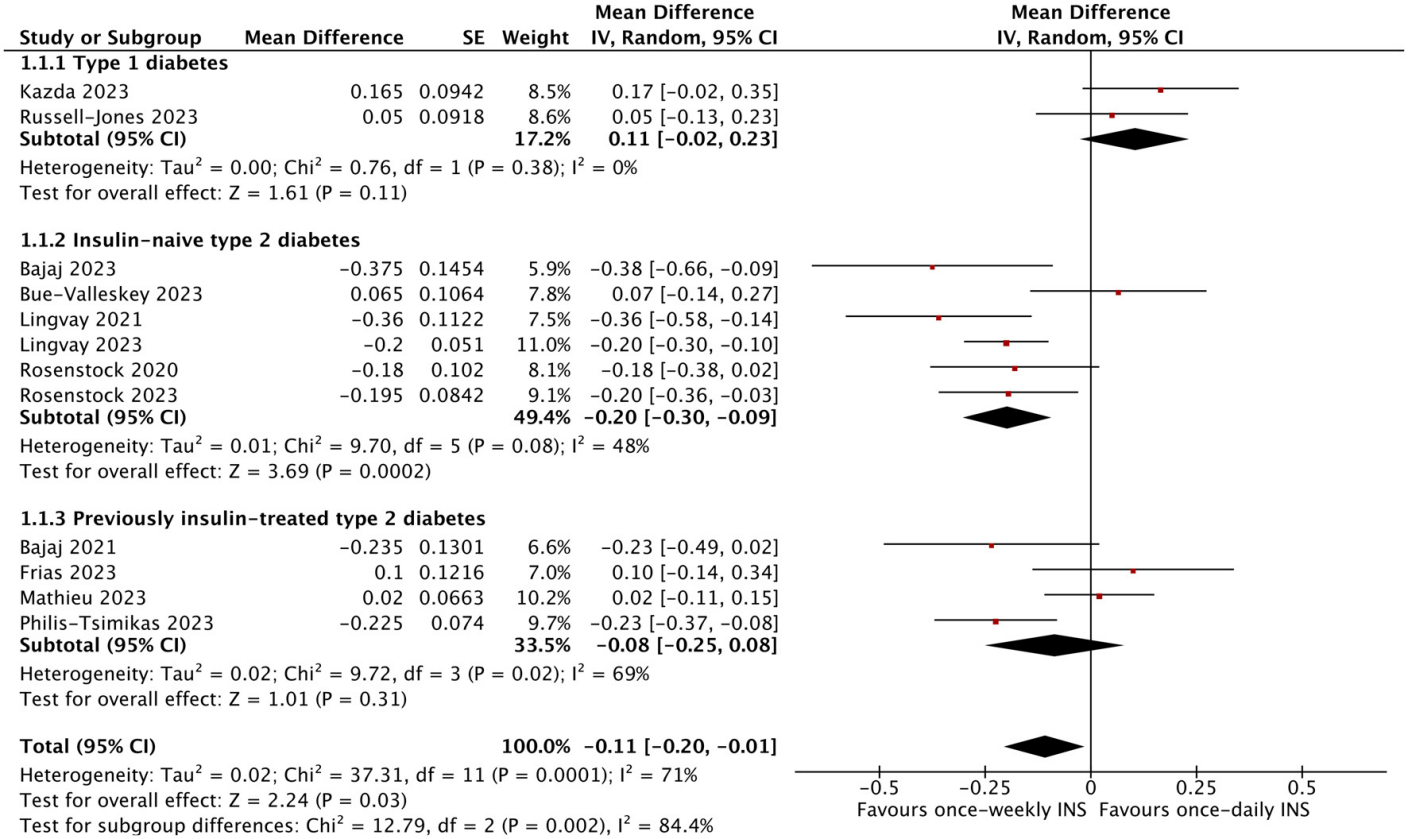
The forest plot of once-weekly insulin vs. once-daily insulin for HbA1c.

In total, the reduction in HbA1c was greater in the once-weekly insulin group (MD −0.11%; 95% CI −0.20 to −0.01%; *I* ^2^ = 71%; *p* = 0.03) compared to the once-daily insulin group. However, the once-weekly insulin intervention only affected the insulin-naïve type 2 diabetic population (MD −0.20%; 95% CI −0.30 to −0.09%; *I* ^2^ = 48%; *p* = 0.0002), and no effect was detected in type 1 diabetes (MD 0.11%; 95% CI −0.02 to 0.23%; *I* ^2^ = 0%; *p* = 0.11) or type 2 diabetes (MD −0.08%; 95% CI −0.25 to 0.08%; *I* ^2^ = 69%; *p* = 0.31), which were previously treated with insulin. The mean change in HbA1c from baseline including all nine trials for the icodec was found to be −0.17% (95% CI −0.26 to −0.08%; *I* ^2^ = 60%; *p* = 0.0002), but there was no significant difference in HbA1c change for BIF (MD 0.12%; 95% CI −0.00 to 0.24%; *I* ^2^ = 0%; *p* = 0.06) (**Figure 3**, **Supplementary Figure 2A**). Moreover, the subgroup analysis based on trial duration indicated that the duration of treatment between the two groups was not associated with a reduction in HbA1c (**Supplementary Figure 2B**). The subgroup analysis based on diabetes duration revealed that a duration below the median was associated with a significantly greater reduction in HbA1c (MD −0.20%; 95% CI −0.30 to −0.09%; *I* ^2^ = 48%; *p* = 0.0002) (**Supplementary Figure 2C**). When the comparator insulin was the same, the analysis separately showed that the HbA1c reduction was −0.17% versus glargine (95% CI −0.31 to −0.04%; *I* ^2^ = 63%; *p* = 0.01) and did not reveal a significant difference versus degludec (MD −0.02%; 95% CI −0.17 to 0.12%; *I* ^2^ = 71%; *p* = 0.76) (**Supplementary Figure 2D**).

Similarly, the pooled odds of HbA1c < 7% at the end of the trial were significantly higher in the intervention group compared with the control group (OR 1.41; 95% CI 1.13 to 1.77; *I* ^2^ = 69%, *p* = 0.003). Subgroup analyses revealed that for the once-weekly insulin intervention, more events of achieving an end-of-trial HbA1c < 7% occurred in insulin-naïve type 2 diabetic participants (OR 1.67; 95% CI 1.44 to 1.94; *I* ^2^ = 0%, *p* < 0.00001) or in those who were injected with icodec (OR 1.43; 95% CI 1.12 to 1.82; *I* ^2^ = 72%, *p* = 0.004) (**Supplementary Figure 3**).

**Figure 4:**
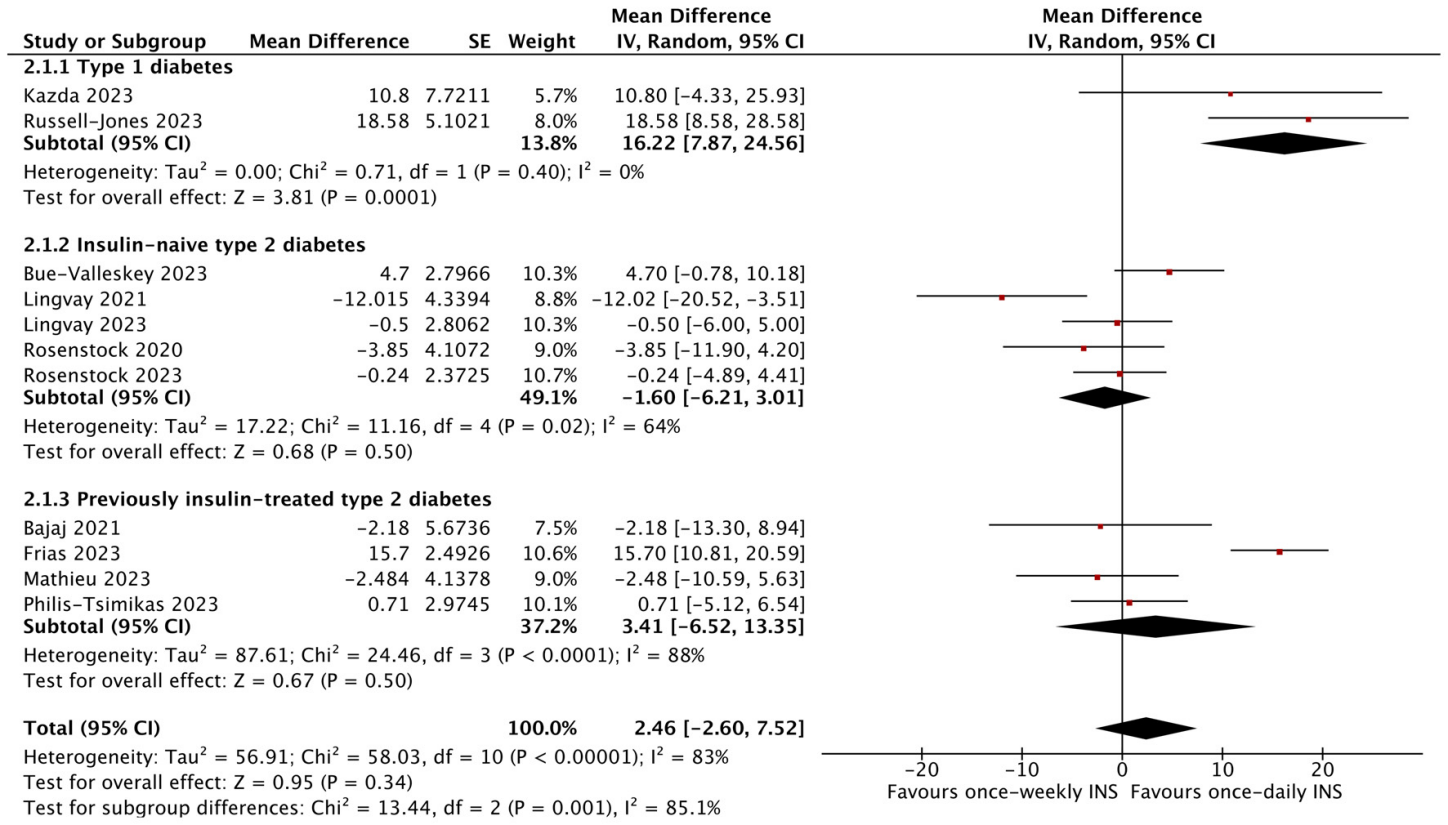
The forest plot of once-weekly insulin vs. once-daily insulin for fasting plasma glucose (FPG).

However, no effect on FPG (MD 2.46 mg/dL; 95% CI −2.60 to 7.52 mg/dL; *I* ^2^ = 83%, *p* = 0.34) and TIR (MD 2.03%; 95% CI −0.50 to 4.56%; *I* ^2^ = 77%, *p* = 0.12) was noted for the entire diabetic population between the two groups. Specifically, TIR was significantly higher with once-weekly insulin than with once-daily insulin in insulin-naïve type 2 diabetics (MD 4.57%; 95% CI 2.63 to 6.51%; *I* ^2^ = 0%, *p* < 0.00001) and icodec-treated patients (MD 2.78%; 95% CI 0.27 to 5.30%; *I* ^2^ = 74%, *p* = 0.03) (**Figure 4**, **Supplementary Figure 4**).

Furthermore, a slight weight gain was observed with once-weekly insulin compared to once-daily insulin (MD 0.43 kg; 95% CI 0.09 to 0.76 kg; *I* ^2^ = 43%, *p* = 0.01). When assessed separately, patients with insulin-naïve type 2 diabetes (MD 0.45 kg; 95% CI 0.13 to 0.77 kg; *I* ^2^ = 0%, *p* = 0.006) or treated with icodec (MD 0.54 kg; 95% CI 0.26 to 0.81 kg; *I* ^2^ = 4%, *p* = 0.0001) had a significant weight gain compared to once-daily insulin (**Supplementary Figures 5A, B**). Importantly, the once-weekly insulin group gained more weight than the control group when the trial by Frias with weight data that differed significantly from other trials was excluded (MD 0.53 kg; 95% CI 0.27 to 0.78 kg; *I* ^2^ = 0%, *p* < 0.0001) (**Supplementary Figure 5C**).

### 3.5 Safety outcomes

**Figure 5:**
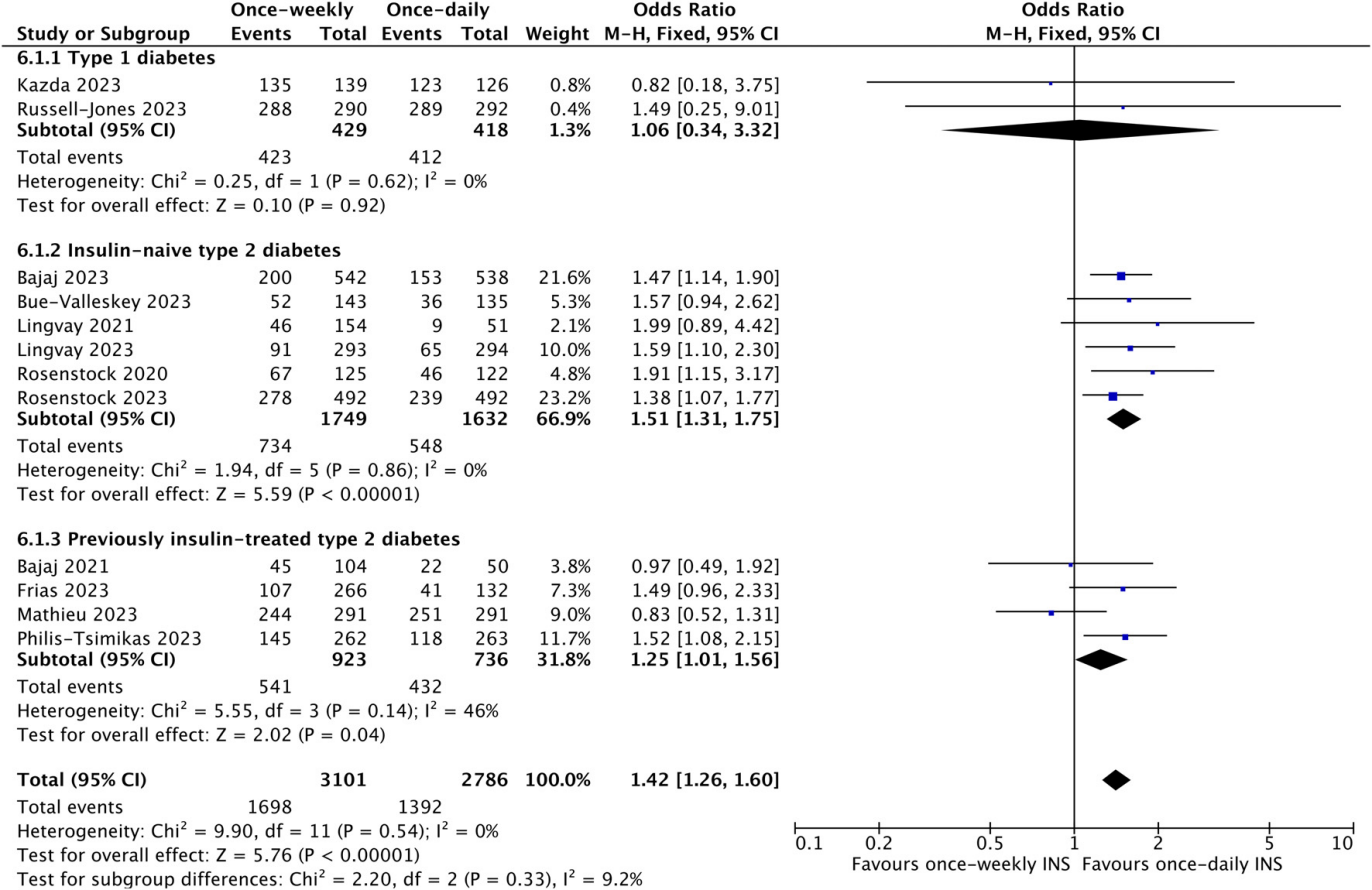
The forest plot of once-weekly insulin vs. once-daily insulin for level 1 hypoglycemic events.

**Figure 6:**
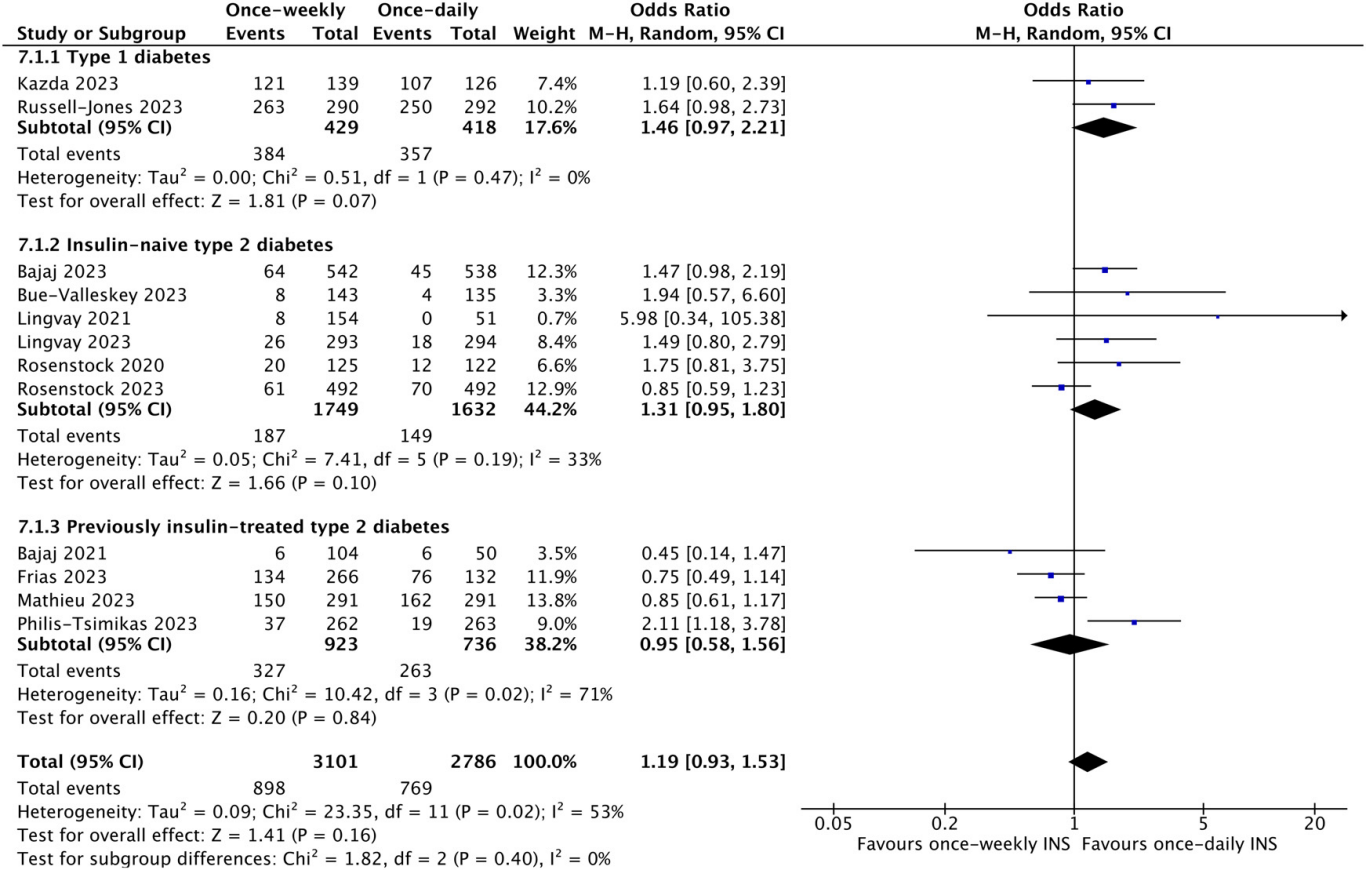
The forest plot of once-weekly insulin vs. once-daily insulin for level 2 or 3 hypoglycemic events.

In terms of safety endpoints, the results showed significantly higher rates of level 1 hypoglycemia with once-weekly insulin (OR 1.42, 95% CI 1.26 to 1.60; *I* ^2^ = 0%; *p* < 0.00001). Subgroup analysis showed an increased risk of level 1 hypoglycemia in patients with type 2 diabetes regardless of previous insulin use (for insulin-naïve type 2 diabetes, OR 1.51, 95% CI 1.31 to 1.75; *I* ^2^ = 0%; *p* < 0.00001; for non-insulin-naïve type 2 diabetes, OR 1.25, 95% CI 1.01 to 1.56; *I* ^2^ = 46%; *p* = 0.04) and regardless of the type of insulin used (for icodec, OR 1.41, 95% CI 1.24 to 1.61; *I* ^2^ = 13%; *p* < 0.00001; for BIF, OR 1.48, 95% CI 1.07 to 2.05; *I* ^2^ = 0%; *p* = 0.02) (**Figure 5**, **Supplementary Figure 6A**). However, clinically significant (level 2) or severe (level 3) hypoglycemia was not significantly different in the once-weekly insulin group compared with once-daily insulin (OR 1.19; 95% CI 0.93 to 1.53; *I* ^2^ = 53%; *p* = 0.16) (**Figure 6**, **Supplementary Figure 6B**).

In addition, once-weekly insulin treatment was associated with an 18% increased incidence of any adverse event (OR 1.18; 95% CI 1.06 to 1.32; *I* ^2^ = 0%; *p* = 0.003) and a 24% increased risk of any adverse event probably or possibly related to basal insulin (OR 1.24; 95% CI 1.03 to 1.50; *I* ^2^ = 18%; *p* = 0.02) (**Supplementary Figure 7**). Nevertheless, once-weekly insulin therapy did not increase the risk of serious adverse events (OR 0.93; 95% CI 0.77 to 1.13; *I* ^2^ = 0%; *p* = 0.48), severe adverse events (OR 0.98; 95% CI 0.76 to 1.28; *I* ^2^ = 47%; *p* = 0.91), injection-site reactions (OR 1.30; 95% CI 0.90 to 1.89; *I* ^2^ = 0%; *p* = 0.17), and hypersensitivity events (OR 1.00; 95% CI 0.78 to 1.29; *I* ^2^ = 2%; *p* = 0.97) (**Supplementary Figures 8**, **9**).

## 4 Discussion

### 4.1 Main findings

As we all know, optimal glycemic management is the cornerstone of reducing the risk of diabetic complications. While the majority of patients with type 2 diabetes initially start with oral hypoglycemic drugs, eventually many will need insulin therapy. Timely and effective use of basal insulin is essential for glycemic management and prevention of complications in patients with type 2 diabetes (28–30). Studies comparing once-weekly and once-daily Glucagon-Like Peptide-1 (GLP-1) receptor agonists have shown that once-weekly injections can achieve non-inferior reductions in HbA1c and weight loss, in addition to higher patient compliance and satisfaction (31–33). Combining the once-weekly GLP-1 receptor agonists with glucose-dependent insulinotropic peptide or the long-acting amylin analog resulted in clinically relevant improvements in glycemic control and weight loss in type 2 diabetics (34, 35). Therefore, the shift in insulin injection mode from once a day to once a week or even once a month is also a general trend. In the future, the combination of once-weekly basal insulin with once-weekly GLP-1 receptor agonists may provide better benefits for diabetic patients. Icodec and BIF are two once-weekly basal ultra-long insulin analogs through special modifications (5). In this study, we aimed to estimate the efficacy and safety of once-weekly insulin (icodec and BIF) vs. once-daily insulin (glargine and degludec) in type 1 and type 2 diabetic patients who were either insulin-naïve or already receiving insulin treatment with or without oral glucose-lowering agents.

Our results are similar to previous systematic reviews and meta-analyses that have shown that once-weekly insulin is superior to once-daily insulin for glycemic control in terms of HbA1c in type 2 diabetes. Nevertheless, neither these studies nor ours found differences in fasting glucose control between the two groups. With regard to TIR, there was no difference in the overall effect, but other studies have revealed higher TIR in the once-weekly insulin group in which only studies on icodec were included (15–17, 19).

In contrast to the above-published articles, our analysis included trials with a study population of type 1 diabetes and included the largest number of articles. In the study by Ribeiro, diabetic patients who have previously been treated with insulin will have a change in their response to the replacement insulin therapy, thus affecting the results of the analysis (16). We tried to compare the efficacy and safety of once-weekly versus once-daily insulin in individuals with different types of diabetes or whether they had been treated with insulin in the past. Therefore, we performed a subgroup analysis according to the different types of participants including type 1 diabetes, insulin-naïve type 2 diabetes, and previously insulin-treated type 2 diabetes. Vora et al. made the same subgroup classification to obtain the differences between insulin degludec and glargine (36). Similarly, subgroup classifications based on the characteristics of the participants, such as insulin resistance and mixed population, were used to evaluate the efficacy of GLP-1 receptor agonists in children with obesity (37). Our study showed a greater HbA1c reduction and higher TIR in insulin-naïve type 2 diabetics, whereas there was no difference in subjects who had used insulin previously, regardless of the type of diabetes. The conclusion of the subgroup analysis may help us to target the use of once-weekly insulin in specific populations for greater glycemic control benefits.

Currently, no trials have directly compared the efficacy and safety of icodec and BIF in diabetic patients. Karakasis et al. found that compared with once-daily analogs, greater reduction in HbA1c levels caused by once-weekly basal insulin was attributed to insulin icodec, while BIF showed nonsignificant differences through subgroup analysis (15). Recently, a network meta-analysis was conducted to compare their relative effectiveness, and the results showed significantly higher efficacy of icodec compared to BIF in type 2 diabetes (18). Consistently, our subgroup analysis also indirectly suggested that icodec could achieve better HbA1c reduction and higher TIR than BIF.

Weight gain and hypoglycemia, common side effects of insulin therapy, may delay its initiation and intensification (38). Once-weekly basal insulin resulted in an additional 0.43 kg increase in body weight as compared to once-daily insulin, especially in insulin-naïve type 2 diabetic and icodec-treated patients, which was consistent with studies of Mukhopadhyay and Abuelazm (17, 19). In the trial by Frias, BIF caused less weight gain compared to degludec, which was different from the results of the study by Bue-Valleskey (10, 22). This may be related to the participants’ previous use of basal insulin and three oral antidiabetic medications, along with their lower fasting blood glucose at baseline. Unfortunately, the average dosage of BIF was not reported in the trial by Frias, and we were unable to compare the insulin dosages of patients in the two trials. The number of current studies is small, and more RCTs are needed in the future to explore this pending issue. The once-weekly insulin group gained more weight (0.53 kg) than the control group when the data from the trial by Frias were exclued, whereas the weight gain was 0.43 kg when the data from this trial were included. Overall, the results of the body weight analysis were consistent. Lifestyle changes such as diet and exercise can mitigate insulin-induced weight gain, especially in patients with diabetes who are already overweight (38). Obesity-related insulin resistance, metabolic syndrome, and type 2 diabetes are closely associated (39). For these patients, insulin therapy can be combined with hypoglycemic drugs, such as sodium-glucose cotransporter-2 inhibitors and GLP-1 receptor agonists, to help lose weight and optimize glycemic control (40, 41).

Regarding safety outcomes, although the risk of level 1 hypoglycemia was increased by 42% with once-weekly insulin injections, the risk of clinically significant or severe hypoglycemia was similar to that with once-daily insulin injections. Moreover, once a week insulin therapy slightly increased the risk of any adverse event and any adverse event probably or possibly related to basal insulin. However, once-weekly insulin was not associated with the risk of serious adverse events, severe adverse events, injection-site reactions, and hypersensitivity events compared to once-daily insulin. These results for safety endpoints in our study are similar to previous studies (15, 17).

### 4.2 Limitations

There are several potential limitations of the study that should be considered. First, the number of RCTs included in the analysis was small. Second, the trial duration and titration algorithms among the included trials varied, which may lead to heterogeneity. Furthermore, only two trials used blind designs and most were open-label, which may affect the adjustment of insulin dose and the reporting or monitoring of adverse events. Finally, we did not compare the differences between the two interventions in terms of insulin dose, nocturnal hypoglycemia, or the number of repeated hypoglycemia in the same individual, because few relevant data were reported in most trials. However, we do not believe that any of these limitations will change the conclusions of this review.

### 4.3 Conclusion

In conclusion, this study demonstrated that once-weekly insulin achieved superior HbA1c control compared to once-daily insulin, with no significant differences in clinically significant or severe hypoglycemic events, although the risk of weight gain and level 1 hypoglycemia events was slightly increased. In addition, once-weekly insulin was associated with equivalent FPG levels and TIR compared with once-daily insulin. The above findings were mainly observed in insulin-naïve type 2 diabetic participants or those who were injected with icodec. The results suggested that when patients with type 2 diabetes first initiate insulin therapy, once-weekly insulin icodec may be the preferred treatment option. Further RCTs are needed to directly compare the effects of the two once-weekly insulins in different types of diabetes.

## Data Availability

The original contributions presented in the study are included in the article/**Supplementary Material**. Further inquiries can be directed to the corresponding authors.
