# Supplementary material for: Efficacy and safety of once-weekly insulin versus once-daily insulin in patients with type 1 and type 2 diabetes mellitus: an updated meta-analysis of randomized controlled trials
Source: Front Endocrinol (Lausanne). 2024 Nov 19;15:1459127. doi: 10.3389/fendo.2024.1459127 (PMC11611561; doi:10.3389/fendo.2024.1459127)
Supplement: Supplementary file 1 [file DataSheet1.docx]

Supplementary Material

Efficacy and safety of once-weekly insulin versus once-daily insulin in patients with type 1 and type 2 diabetes mellitus: An updated meta-analysis of randomized controlled trials

Mei Xue^1#^, Pan Shen^2#^, Jun Tang^1^, Xuan Deng^3*^, Zhe Dai^1*^.

^1^Department of Endocrinology, Zhongnan Hospital of Wuhan University, 430071 Wuhan, China.

^2^Department of Dermatology, Tongji Hospital, Tongji Medical College of Huazhong University of Science and Technology, 430030 Wuhan, China.

^3^Department of Nephrology, Zhongnan Hospital of Wuhan University, 430071 Wuhan, China.

^#^These authors have contributed equally to this work.

*Corresponding authors:

Zhe Dai. daizhe@znhospital.cn.

Xuan Deng. 761237742@qq.com.

# Supplementary Tables

## Supplementary Table 1

Supplementary Table 1. Search strategy for Pubmed.

| #1 | "once-weekly basal insulin" OR "once weekly basal insulin" OR "once-weekly insulin" OR "once weekly insulin" OR "Insulin icodec" OR "Icodec" OR "Basal insulin Fc" OR "BIF" OR "Insulin efsitora alfa" OR "LY3209590" |
| --- | --- |
| #2 | "randomized controlled trial"[pt] OR "controlled clinical trial"[pt] OR randomized[tiab] OR placebo[tiab] OR "drug therapy"[sh] OR randomly[tiab] OR trial[tiab] OR groups[tiab] |
| #1 AND #2 | Results: 200 |

## Supplementary Table 2

Supplementary Table 2. Search strategy for Embase.

| #1 | "once-weekly basal insulin" OR "once weekly basal insulin" OR "once-weekly insulin" OR "once weekly insulin" OR "Insulin icodec" OR "Icodec" OR "Basal insulin Fc" OR "BIF" OR "Insulin efsitora alfa" OR "LY3209590" |
| --- | --- |
| #2 | 'randomized controlled trial'/exp OR 'controlled clinical trial'/exp OR randomized:ti,ab OR placebo:ti,ab OR 'drug therapy':lnk OR randomly:ti,ab OR trial:ti,ab OR groups:ti,ab |
| #1 AND #2 | Results: 373 |

## Supplementary Table 3

Supplementary Table 3. Search strategy for Cochrane Library.

| #1 | "once-weekly basal insulin" OR "once weekly basal insulin" OR "once-weekly insulin" OR "once weekly insulin" OR "Insulin icodec" OR "Icodec" OR "Basal insulin Fc" OR "BIF" OR "Insulin efsitora alfa" OR "LY3209590" |
| --- | --- |
|  | Results: 206 (206 Trials and 5 Cochrane Reviews) |

# Supplementary Figures

## Supplementary Figure 1


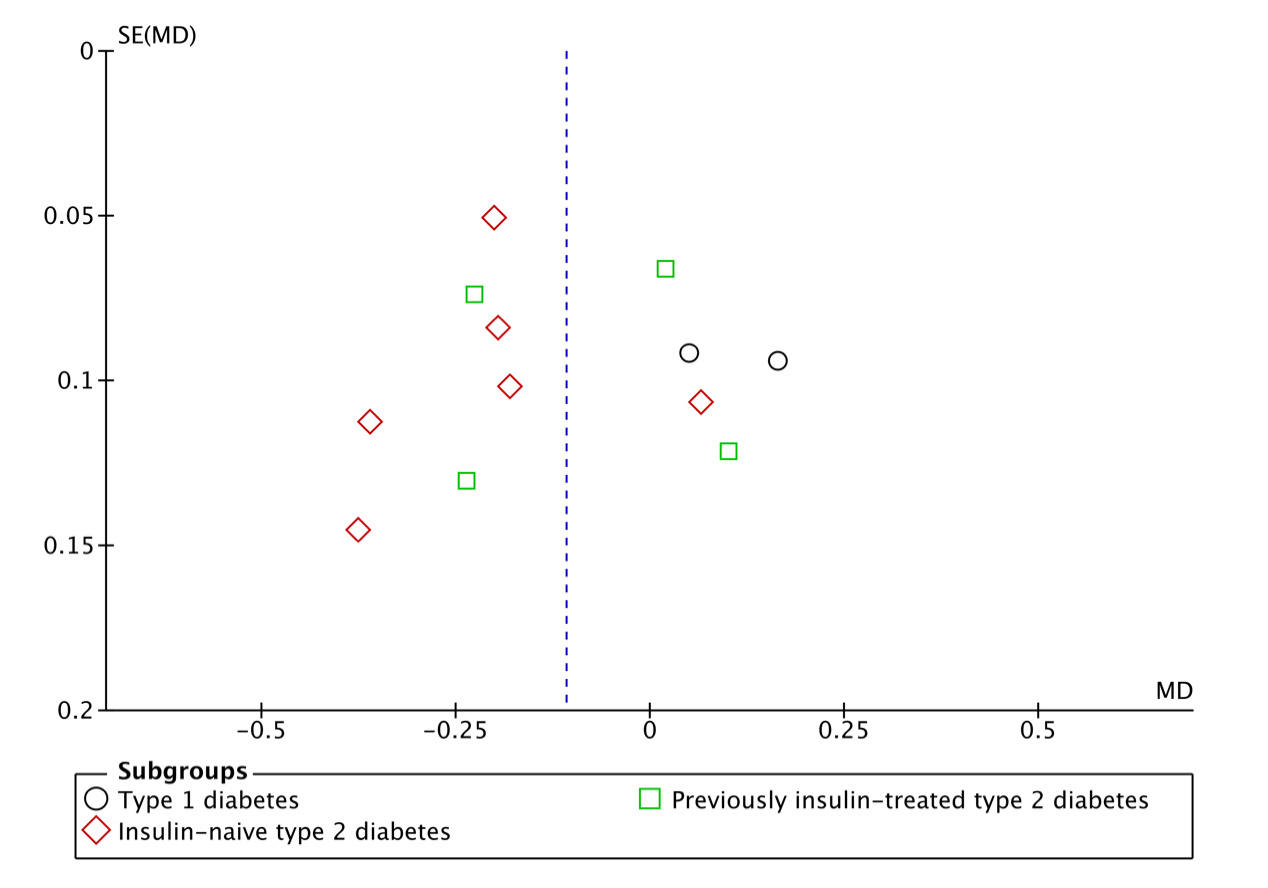


Supplementary Figure 1. Publication bias detected by funnel plots for HbA1c.

## Supplementary Figure 2


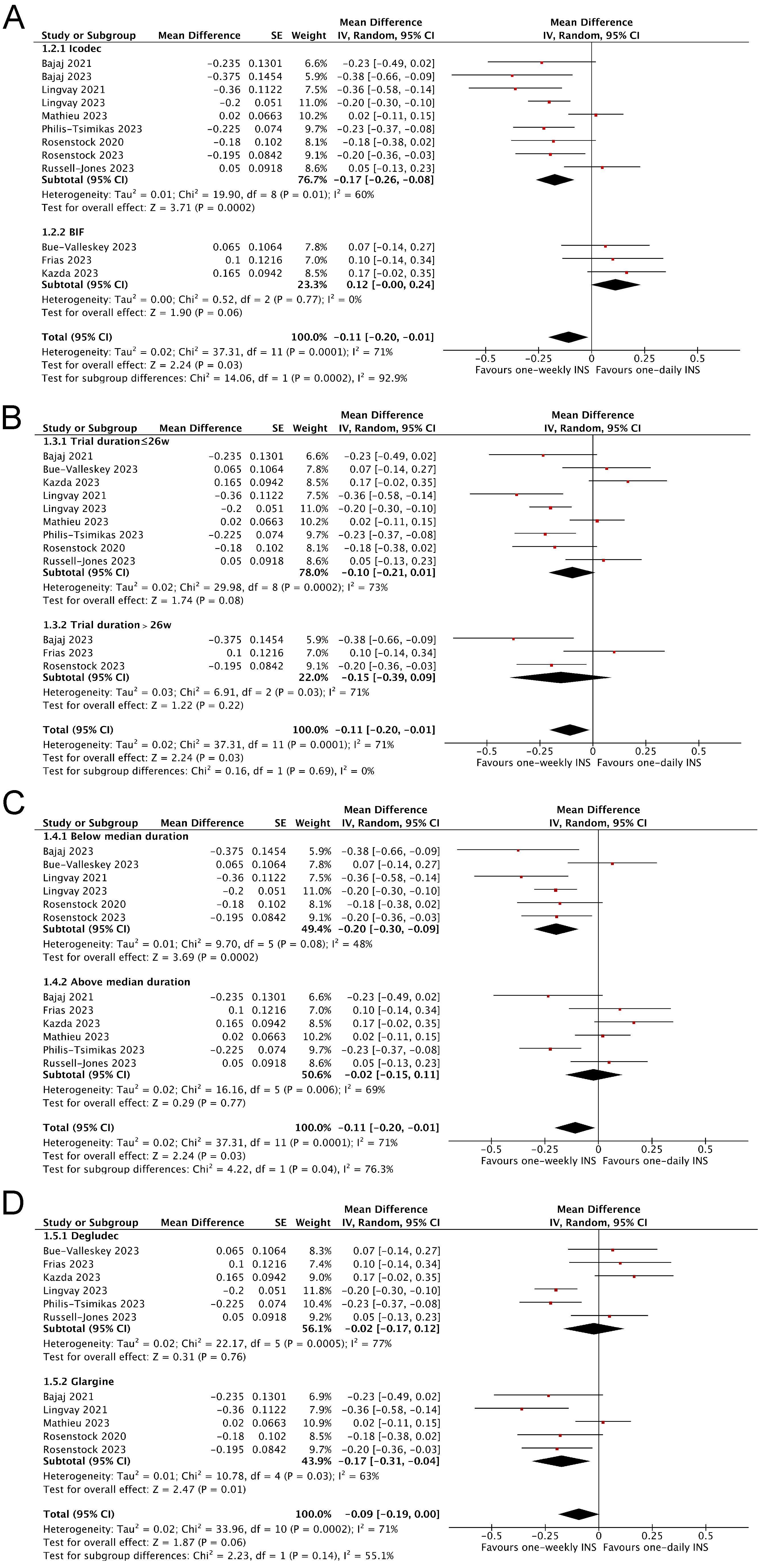


Supplementary Figure 2. The forest plot of once-weekly insulin vs. once-daily insulin for HbA1c. Subgroup analyses were based on types of once-weekly insulin (A), trial duration (B), diabetes duration (C), and types of once-daily insulin (D).

## Supplementary Figure 3


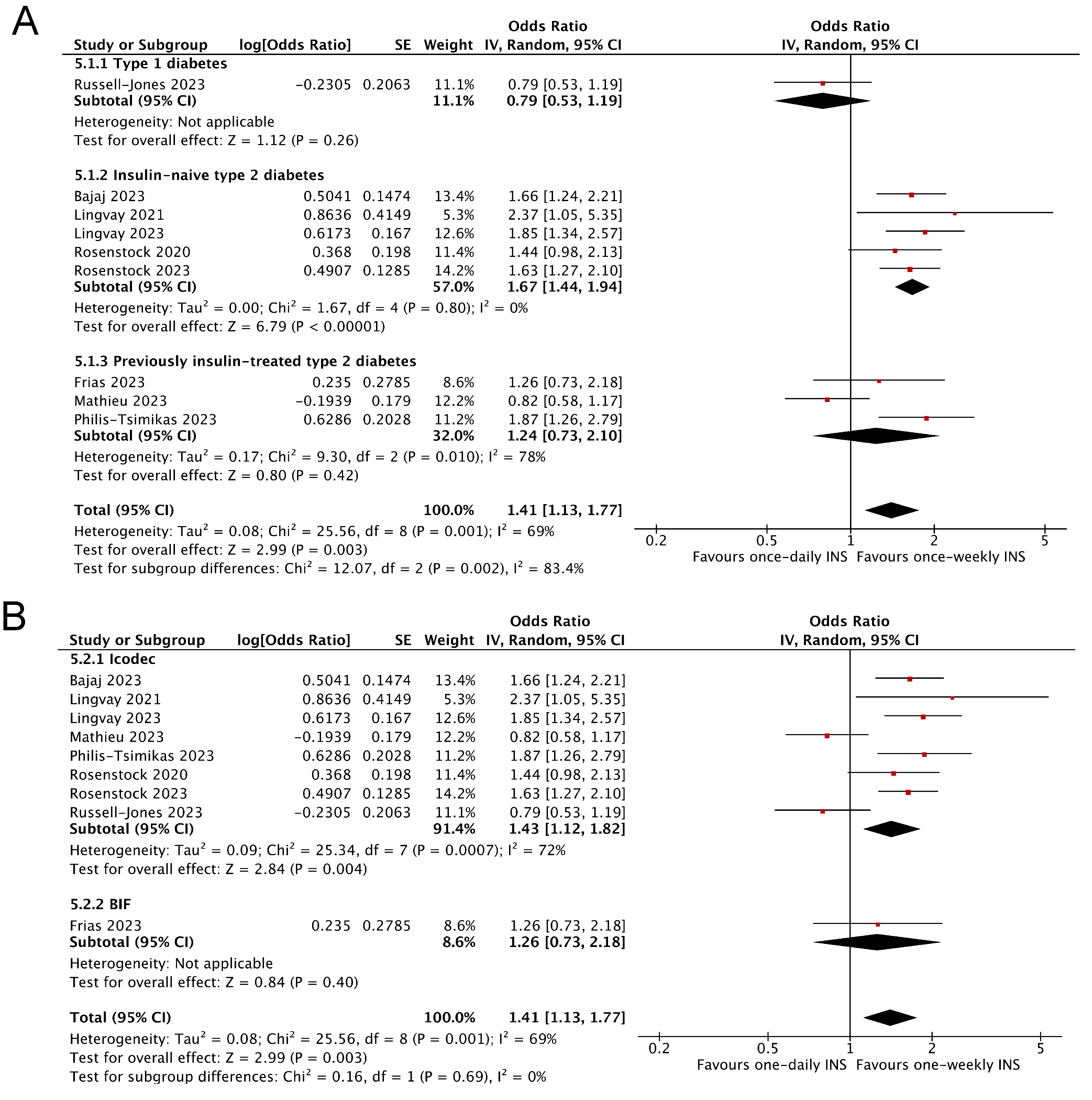


Supplementary Figure 3. The forest plot of once-weekly insulin vs. once-daily insulin for an odds ratio of patients achieving an end-of-trial ΗbΑ1C < 7%. Subgroup analyses were based on different types of participants (A), and types of once-weekly insulin (B).

## Supplementary Figure 4


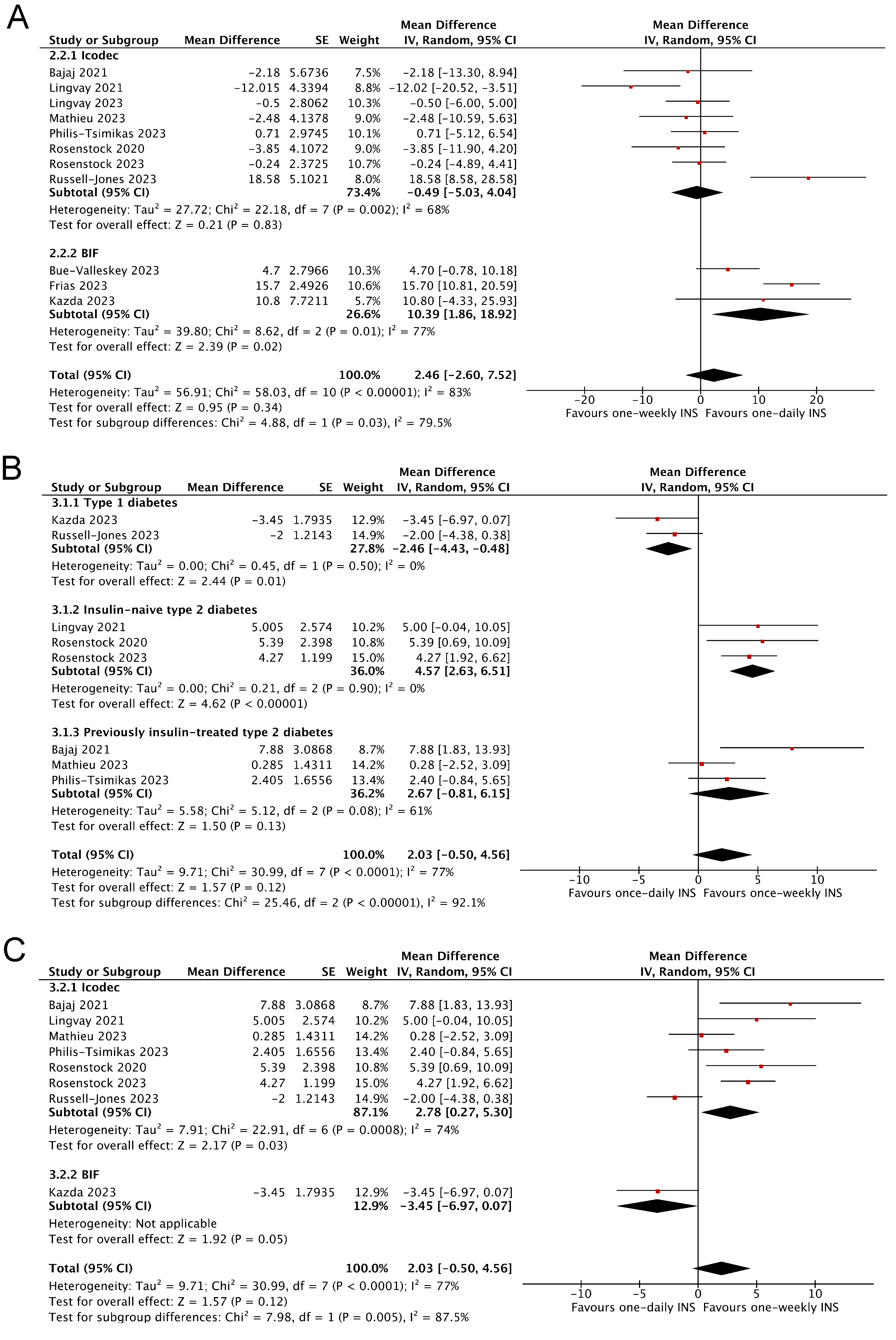


Supplementary Figure 4. The forest plot of once-weekly insulin vs. once-daily insulin for fasting plasma glucose (FPG) (A) and time in range (TIR) (B, C). Subgroup analyses were based on different types of participants (B), and types of once-weekly insulin (A, C).

## Supplementary Figure 5


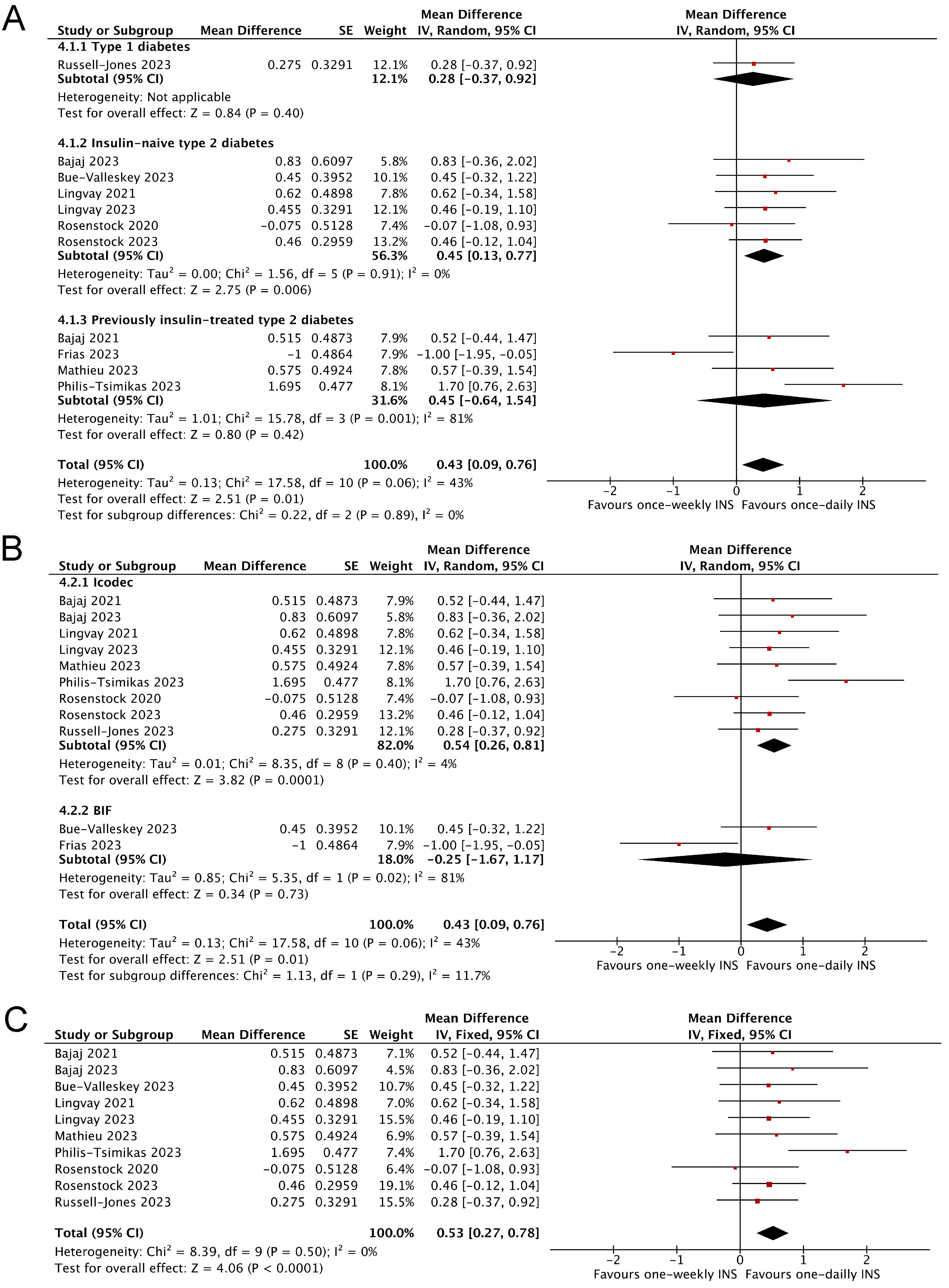


Supplementary Figure 5. The forest plot of once-weekly insulin vs. once-daily insulin for body weight. Subgroup analyses were based on different types of participants (A), and types of once-weekly insulin (B). Analysis of the data excluding the Frias 2023 trial (C).

## Supplementary Figure 6


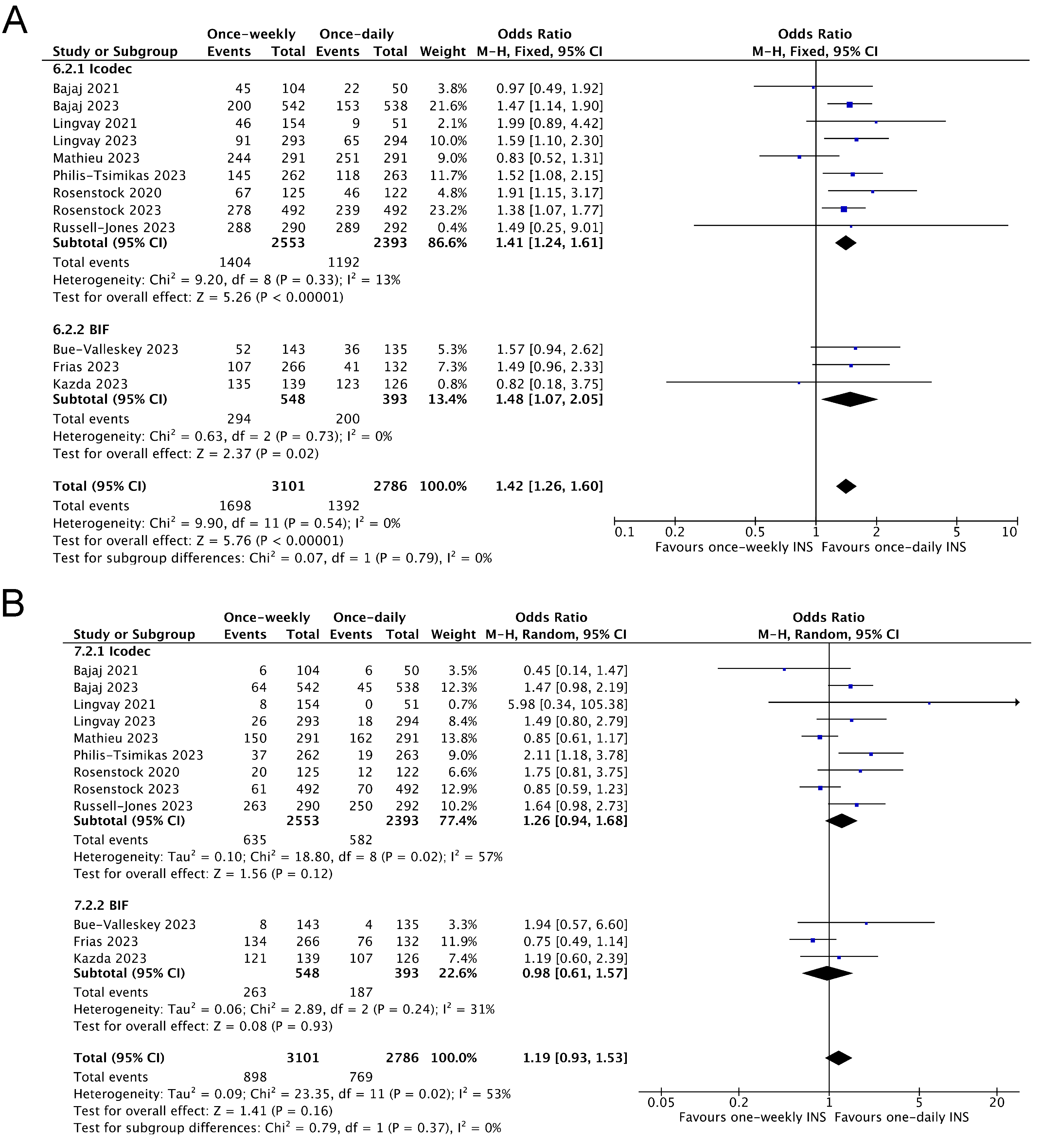


Supplementary Figure 6. The forest plot of once-weekly insulin vs. once-daily insulin for level 1 (A) and level 2 or 3 (B) hypoglycemic events. Subgroup analyses were based on types of once-weekly insulin.

## Supplementary Figure 7


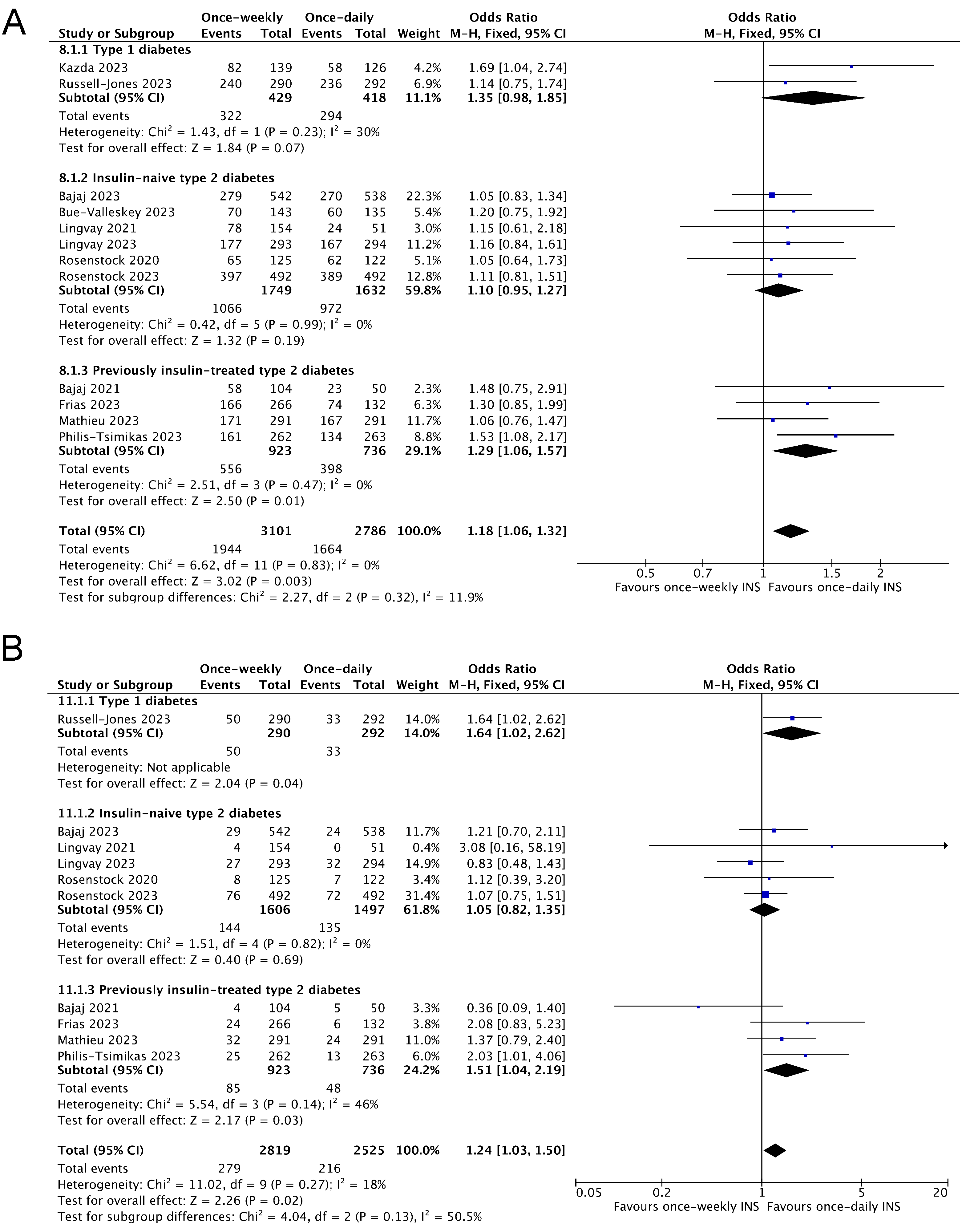


Supplementary Figure 7. The forest plot of once-weekly insulin vs. once-daily insulin for any adverse event (A) and any adverse event probably or possibly related to basal insulin (B). Subgroup analyses were based on different types of participants.

## Supplementary Figure 8


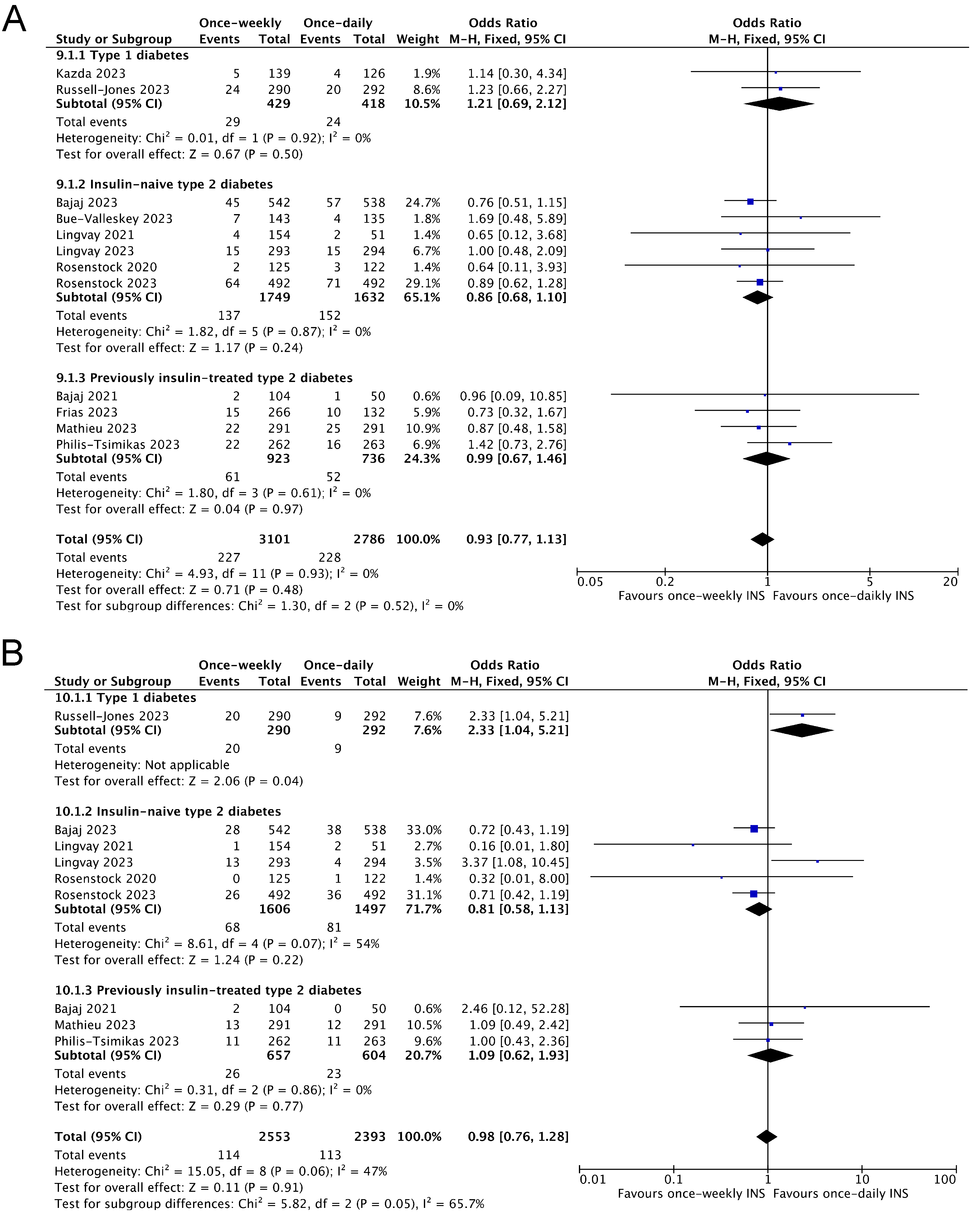


Supplementary Figure 8. The forest plot of once-weekly insulin vs. once-daily insulin for serious adverse events (A) and severe adverse events (B). Subgroup analyses were based on different types of participants.

## Supplementary Figure 9


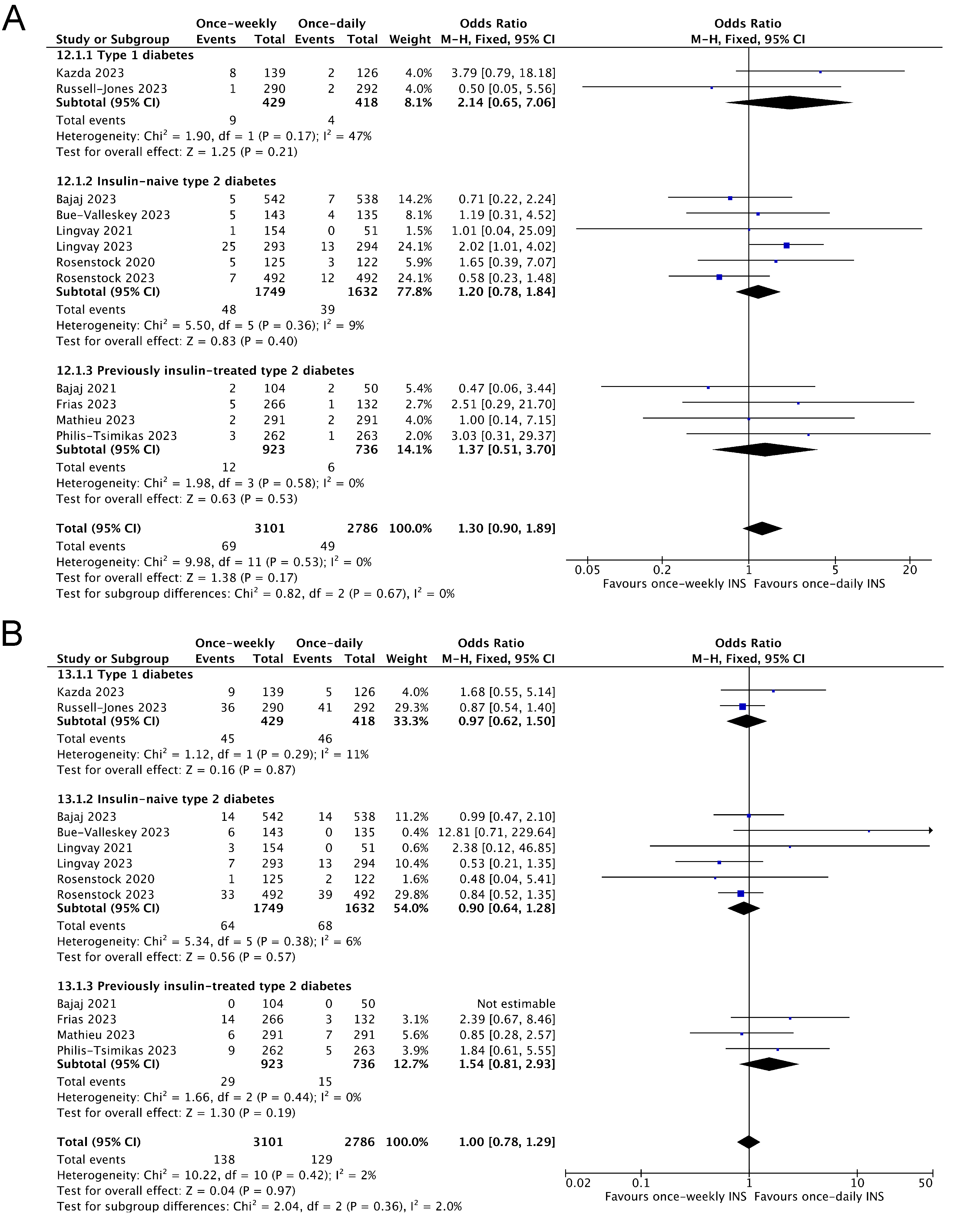


Supplementary Figure 9. The forest plot of once-weekly insulin vs. once-daily insulin for injection-site reaction (A) and hypersensitivity events (B). Subgroup analyses were based on different types of participants.
